# Six Years of Panel Practice

**Published:** 1918-12-21

**Authors:** J. C. Lyth

**Affiliations:** Member of Council Panel, Medico-Polit. Union.


### SIX YEARS OF PANEL PRACTICE.

### Advantages, Disadvantages, and the Remedies.

By J. C. LYTH, M.B., B.S. Lond., Member of Council Panel, Medico-Polit. Union.

We have now almost completed the sixth year following the institution of medical benefit under the National Health Insurance Act, and the amending Acts. Of this time more than four years have been spent under the stress of war; and while this has in some ways vitiated the period as a test of panel practice, it has served to bring out the more rapidly some of the essential characteristics of the system.

The war is practically over; the spirit of reconstruction is in the air. A Bill for the constitution of a Ministry of Health, with wide powers and expressly to supersede the National Health Insurance Commission, is ready for introduction at the coming Session of Parliament. It is well, therefore, for practitioners working under the Acts to take stock, to count up their gains and their losses, and to determine whether, and if so under what conditions, it may be worth their while to continue as servants of the State.

I propose then (very briefly in the space at my disposal) to discuss the advantages and the disadvantages which an average general practitioner feels to have been introduced to him by the working of the Acts, and to endeavour to show in what ways the principal grievances felt by the panel practitioners may be removed.

### Advantages.

1. A settled income is provided which can be calculated approximately in advance.

2. As a result of this, but for the war, the ease of sale and the market value of practices would have been increased.

3. There is increased ease in introducing a substitute in case of illness or other cause of absence.

4. There is freedom from dispensing (in urban practice) and in sending out of accounts.

5. There are fixed rules for patients (unfortunately honoured largely in the breach).

These advantages are not inconsiderable, and some of them have been brought into prominence by war conditions. For example, panel practitioners on war service have been drawing half their panel fees regularly every quarter; further, with the extreme shortage of doctors, it would have been quite impossible to deal with the influenza epidemic had it been necessary to dispense the medicines.

On the debit side, however, the disadvantages loom big, and, as we shall see, some of them are in direct contrast to the promises held out when the system was introduced.

### Disadvantages.

1. *Remuneration.*—The capitation fee is less than we had been led to expect; and the payment for work done is actually decreasing, and is relatively much less than for private practice.

In 1912 it was stated (I quote from the official pamphlet) that for every 1,000 persons *on our lists* for the year we should receive at least £325; with the tuberculosis 6d. and the 6d. from the Drug Suspense Fund, £375. Also that we might "confidently rely" on receiving at least 7s. and possibly (with the drug 6d.) 7s. 6d. per head per annum of all persons *on our lists.* Yet I have before me the "Final Settlement" issued in my own area for 1916, to a doctor whose list was 968 for that year; that is to say, that all or any of these 968 individuals were entitled to claim treatment from him. It is there stated that the amount due at 7s. per head would be £338 16s., but that in view of an "inflation" of 20.61 per cent., which is stated to have occurred in the lists, the amount paid would be £268 19s. 7d., a difference of some £70. Moreover, that "Final Settlement" was not made until November 25, 1917.

This doctor offered to verify his list at his own expense, but was informed that such a proceeding would be quite useless, and would not affect the payment. I do not propose to enter again into the legal quibble whereby this gross discrepancy has been justified; I am dealing only with facts, and I say such a state of affairs is most unsatisfactory from the practitioner's point of view, and calls urgently for remedy.

In regard to payment for work done, figures collected in my own area show that the sum paid for each attendance recorded is diminishing year by year, probably chiefly because the number of old and otherwise incapacitated insured persons who remain in medical benefit but for whom no fees are paid, is steadily increasing, and must increase for a generation of working life. The attendance value is now considerably less than in 1913. It is hardly necessary to stress the difference between panel fees and the fees for private practice. Not only have the latter advanced very largely in the amount charged, but the receipts are in still higher ratio from the great diminution of bad debts. The result of these figures is shown remarkably in the receipts of any practitioner having a mixed panel and private practice; where if the two sources of income were as 1 to 1 in 1913 they are now as 1 to 2, or as 1 to 3. How far this condition will change after the war, time only can show, but it seems improbable that money will resume its pre-war purchasing power.

2. *Time Lost in Treating Trivial Ailments and in Dealing with Malingering.*—While this item is necessarily discounted in dealing with payment for work done, it is none the less of an annoying and demoralising character. It is probably inherent in all contract work.

**Six Years of Panel Practice**—(*continued*).

3. *Certification.*—This forms one of the most galling of the petty annoyances of panel practice. Correctly to complete the official forms of certification it is necessary to state that the patient has been examined on the date noted, is suffering from a certain disease, and is incapable of work. This simple matter is rendered extraordinarily complex by the ignorance and downright sheer cussedness of many Approved Society agents, and by the red-tapeism of some of the societies. Certificates are demanded for certain days of the week only; certain diseases are refused recognition; weekly certificates are demanded in chronic cases (*e.g.*, old hemiplegics). With these may be included the remarkable difficulty which many insured persons experience in recovering sick benefit due to them, which re-acts upon the doctor both in his capacity as medical attendant (since it is difficult to make a starving person well) and in the rôle of guide, philosopher and friend which is so often assigned to him by the patient.

4. *Supply of Medicines.*—Contrary to the statement that "an insured person is entitled to obtain any medicine which the doctor attending him thinks necessary for his treatment," the supply of drugs is severely limited, and only by the strictest economy can a panel practitioner hope to escape the penalty of surcharge.

5. *Lay Control.*—It is probable that a degree of lay control in the matter of complaints is necessary to the working of the Acts, but it is none the less a source of irritation to the average general practitioner that he should be liable to answer for his sins (such as the omission to attend immediately whenever called) before a mixed tribunal such as the Medical Service Sub-Committee.

**6. THE SUPREME POWER OF COMMISSIONERS.**

The Acts place the supreme decision on all matters in the hands of the National Health Insurance Commission. The Commission may remove a practitioner from the panel; may deduct any sum from the fees due to him; may make any alteration whatever in any of the regulations governing his work at any time without notice; may alter the method of payment and even, indirectly, the amount of payment; and all these without any right of appeal to any court whatever.

It is unfortunate that the tone and methods of the Commissioners in carrying out the duties assigned to them have been such as to bring these facts forcibly before the panel practitioners, and especially those chosen to represent them in any negotiations with the Commissioners. All these powers have been exercised by them at different times; sometimes, as in the case of the regulations for the treatment of discharged soldiers, directly contrary to promises previously made; and have been forced through in spite of the most vigorous protestations of representatives of the practitioners.

Human nature is sadly alike the world over. Place a Prussian in power and you produce a savage bully; substitute a Civil servant, and you create an Insurance Commissioner. Sir E. Cornwall's reply to the request of the British Medical Association and the Medico-Political Union for an increase in the capitation fee stands on record as an instance of how a bureaucrat deals with a learned profession when he has the whip hand.

7. *The Agreement is not a Legal Contract.*—As might be deduced from the above remarks, the agreement under which panel practitioners work is not (according to the advice of eminent Counsel) a legal contract, and cannot be enforced at law by either party. But since the Commissioners have the wherewithal to pay the piper, they have the power to call the tune; it is given to the doctors to perform the dance.

Those I think are the principal disadvantages at present attendant upon panel practice. I am not here concerned with defects such as the lack of provision of hospital accommodation, of specialist services, of nursing and the like which are common to all types of medical practice among the working population, and which it is hoped a Ministry of Health will set to work to rectify.

Remedies.

1. In the matter of remuneration it is suggested that the capitation fee should be raised to at least 10s., irrespective of drugs, and that one-fourth of this sum should be paid for each name on the doctor's list at the end of every quarter. If it is necessary to retain a small percentage (say 5 per cent.) for a final settlement, this should be made within a short fixed period after the termination of the war.

2. Malingering (which is markedly less now than formerly owing to high rates of wages obtaining), would be lessened by the appointment of official referees to whom the practitioners, as well as the Societies, might be able to refer difficult patients.

3. Certification should be greatly simplified. Simple fitness or unfitness to follow the occupation should be sufficient, and monthly certificates should be allowed at the doctor's discretion in chronic cases.

4. The money allowed for medicines is insufficient with present prices, and should be increased.

5. The practitioners' agreement should be a legal contract with the right of appeal to common law.

6. Dealings between Government Departments and the panel practitioners should be regulated by the appointment of a Council or Councils, such as are described in the Whitley Report, and foreshadowed in the Ministry of Health Bill. But such Councils must be directly representative and have the confidence of the practitioners concerned.

If some such altered conditions as these are introduced I believe that there is a great future before panel practice. Unless essential reforms are made, I consider that it will cease to attract the better class of general practitioner, and will deteriorate to the level of the old club practice. If the medical profession is sufficiently united to seize the present unique opportunity, nothing within reason is beyond reach, and it is my view that the chief hope of such a consummation lies in the present medical trade union movement. But that is another story.

